# Study on the Influence of Waste Rock Wool on the Properties of Cement Mortar under the Dual Fiber Effect of Polyvinyl Alcohol Fibers and Steel Fibers

**DOI:** 10.3390/ma17143416

**Published:** 2024-07-10

**Authors:** Shijian Lu, Jiajia Cheng, Zhipeng Zhu, Luchao Yan, Yang Wang, Lingling Xu, Min Deng

**Affiliations:** College of Material Science and Engineering, Nanjing Tech University, Nanjing 211816, China

**Keywords:** waste rock wool, fiber-reinforced mortar, mechanical properties, hydration properties, microstructure

## Abstract

In this paper, the effect of waste rock-wool dosage on the workability, mechanical strength, abrasion resistance, toughness and hydration products of PVA and steel fiber-reinforced mortars was investigated. The results showed that the fluidity of the mortar gradually decreased with the increase in the dosage of waste rock wool, with a maximum reduction of 10% at a dosage of 20%. The higher the dosage of waste rock wool, the greater the reduction in compressive strength. The effect of waste rock wool on strength reduction decreases with increasing age. When the dosage of waste rock wool was 10%, the 28 days of flexural and compressive strengths were reduced by 4.73% and 10.59%, respectively. As the dosage of waste rock wool increased, the flexural-to-compressive ratio increased, and at 20%, the maximum value of 28 days of flexural-to-compressive ratio was 0.210, which was increased by 28.05%. At a 5% dosage, the abraded volume was reduced from 500 mm^3^ to 376 mm^3^—a reduction of 24.8%. Waste rock wool only affects the hydration process and does not cause a change in the type of hydration products. It promotes the hydration of the cementitious material system at low dosages and exhibits an inhibitory effect at high dosages.

## 1. Introduction

As a kind of building insulation material, rock wool produces a large number of waste rock wool in the demolition and construction process of buildings. Waste rock wool is expected to grow to 2.82 million tons by 2030 [1]. Taiwan produces over 100 thousand tons of waste rock wool per year [2]. The current treatment of waste rock wool is stacking and landfilling. A large amount of waste rock wool has produced a series of accumulations and environmental problems, so use of waste rock wool urgently needs to be resolved [3,4,5]. In order to reduce the amount of waste rock wool deposited in piles and landfills, Taiwan encourages industries to recycle and reuse waste rock wool with a price subsidy of TWD 1500 per ton [6].

Waste rock wool is an artificial inorganic fiber mainly produced by basalt under high-temperature melting, with high tensile strength, high modulus, chemical resistance and dimensional stability [7,8,9]. Waste rock wool consists of amorphous components such as silica, aluminum oxide and iron trioxide, which are potentially chemically active [10]. Unmilled waste rock wool is reported to have an average diameter of 6–8 microns and a length of 400 microns to 20 mm, and the length of the milled waste rock wool can be less than 34 microns [3,11,12,13,14]. Commonly used construction fibers are steel fibers and PVA fibers. Commonly used steel fibers have a diameter of 200 microns and a length of 13 mm, and commonly used PVA fibers have a diameter of 30 to 40 microns and a length of 6–12 mm. Both commonly used fibers are available in millimeter lengths [14,15]. Thus, waste rock wool, with a diameter of a few micrometers and a length of tens of micrometers, and its potential chemical activity were expected to play a hardening and enhanced role in cement-based materials at the micro-scale. Depending on the particle size and length of the waste rock wool, it can be used as a partial substitute for fine aggregates and as a supplementary cementitious material [16,17,18,19,20]. Many scholars [21,22,23] found that with the increase in mineral cotton content, the formation of large pores in mortar led to an increase in porosity and a general decrease in compressive strength. Kubiliute et al. [24], by collecting the dust from the melting process of a mineral wool raw material (mineral wool cupola dust) used as a cementitious material to replace cement, found that a dosage of washed dust below 20% was able to increase the compressive strength of specimens. Lin et al. [25] found that the incorporation of waste rock wool into cement matrix composites reduces its dry shrinkage and initial surface absorption and improves its compressive strength. These performance improvements were due to the filling effect of waste rock wool and the volcanic ash reaction.

In this paper, based on the optimum volumetric dosage of PVA fibers and steel fibers obtained by the research team, the effect of waste rock wool as a cementitious material and fibrous material on the workability, mechanical strength, toughness and hydration products of PVA fiber and steel fiber mortar was investigated by varying the dosage of waste rock wool. In this study, the volcanic ash properties of waste rock wool were analyzed by X-ray diffraction (XRD), heat of hydration, differential thermal analysis (DSC) and the thermogravimetric method (TG) to assess the effect of waste rock wool on the replacement of Portland cement from a microscopic point of view.

## 2. Experimental Program

### 2.1. Raw Materials

**Figure 1:**
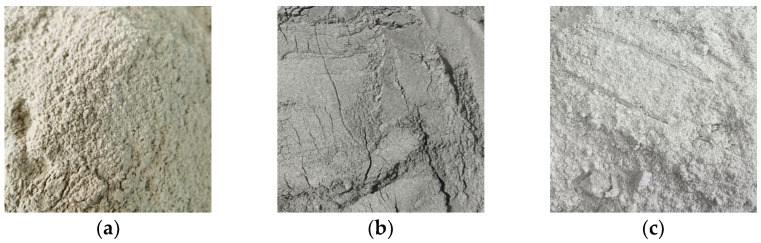
Waste rock wool and supplementary cementitious materials: (**a**) Waste rock wool; (**b**) Fly ash; (**c**) Silica fume.

**Figure 2:**
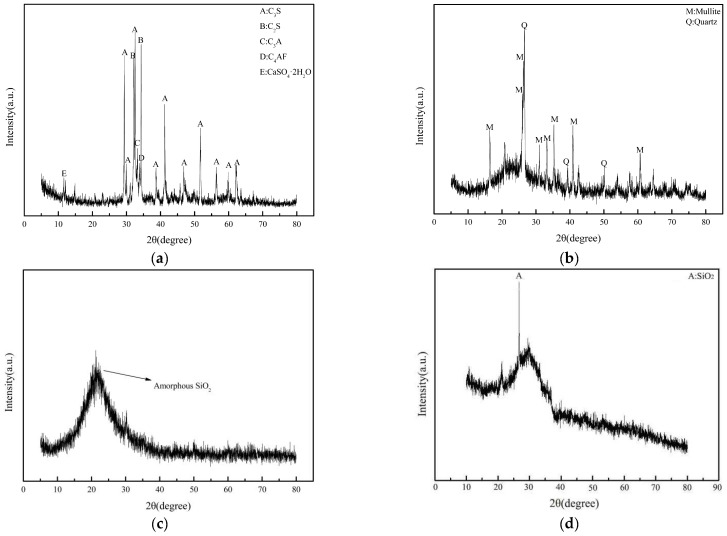
XRD pattern of raw materials: (**a**) Cement; (**b**) Fly ash; (**c**) Silica fume; (**d**) Waste rock wool.

**Figure 3:**
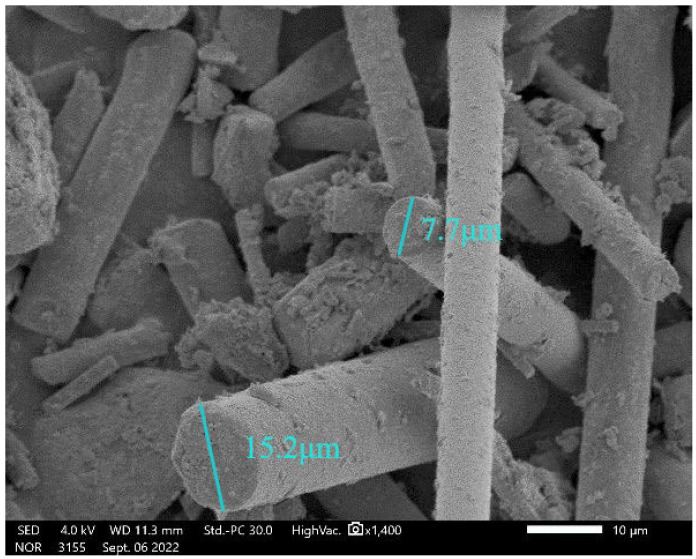
Waste rock-wool SEM image.

The cement used in the test was 52.5-grade ordinary silicate cement (Jiangnan Onoda Cement Co., Nanjing, China). Its 28 days of flexural strength and compressive strength were 8.15 MPa and 54.04 MPa, respectively. Fly ash and silica fume replaced 25% and 5% of cement, respectively. The waste rock wool came from an environmental company in Nanjing, and the particle size ranged from 45 μm to 80 μm. The physical properties of cement are shown in Table 1 [26]. The chemical composition of cement, fly ash and silica fume is shown in Table 2 [26]. Photographs of waste rock wool, fly ash and silica fume are shown in Figure 1. The X-ray diffraction patterns of cement, fly ash, silica fume and waste rock wool are shown in Figure 2. Figure 2b shows that the composition of fly ash was mainly mullite and quartz. Figure 2c shows that silica fume consists of amorphous SiO_2_. Figure 2d shows that the waste rock wool consists of an amorphous substance and SiO_2_. As shown in Figure 3, the waste rock-wool form was rod-shaped. Its diameter was below 20 μm. As shown in Table 3, most of the lengths of waste rock wool were less than 100 μm. The characteristic parameters of PVA fiber and steel fiber are shown in Table 4. The diameter and length of the PVA fiber were 50 μm and 6 mm, and the diameter and length of the steel fiber were 220 μm and 13 mm, respectively.

### 2.2. Mix Design

**Figure 4:**
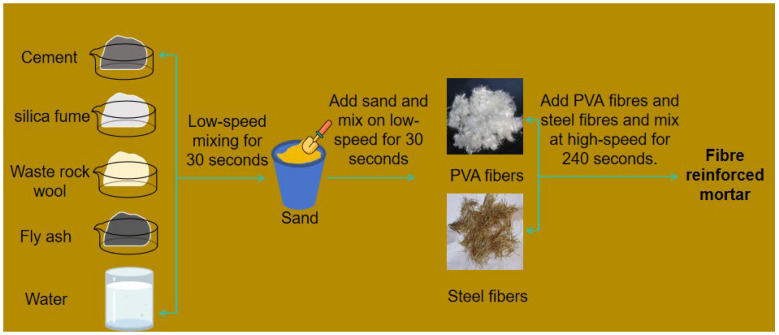
Preparation process of fiber-reinforced mortar.

The sand-to-cement ratio for all mortars specimens was 3:1, with 1350 g of sand and 450 g of cementitious materials. As shown in Table 5, in 450 g of cementitious material, the dosages of fly ash and silica fume were fixed at 25% and 5%, respectively, and the remainder was the dosage of cement. When the dosage of waste rock wool increased by 5%, the dosage of cement was reduced by 5% accordingly. The water/cement ratio was 0.5 s The volumes of polyvinyl alcohol fiber and steel fiber were 0.09% and 0.13% of the mortar specimens volume, respectively. The preparation process of fiber-reinforced mortar is shown in Figure 4. After mixing cement, fly ash, silica fume, water and waste rock wool for 30 s, sand was added with low-speed mixing for 30 s and then high-speed mixing for 60 s, after which steel fibers and PVA fibers were added with low-speed mixing for 240 s to obtain the fiber-reinforced mortar. The mortar was packed into 40 mm × 40 mm × 160 mm molds and cured at 20 ± 2 °C and 95% relative humidity for 24 h, before being cured for 7, 28 and 90 days.

### 2.3. Test Methods

#### 2.3.1. Slurry Fluidity

**Figure 5:**
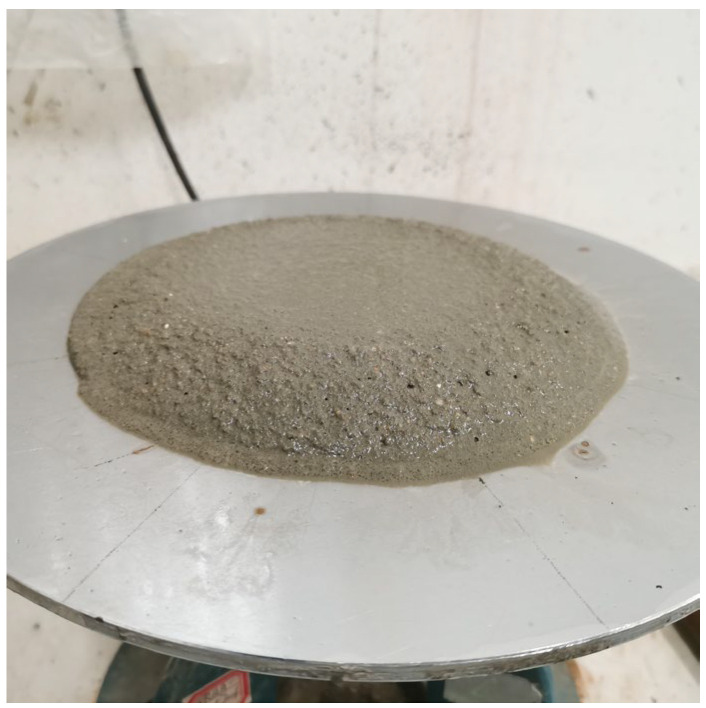
The fluidity test of mortar.

The fluidity test of mortar refers to GB/T 2419-2005 [27]. The fluidity test of mortar is shown in Figure 5. After 25 jumps on the table, we measured the diameter of the mortar in two directions perpendicular to each other and took the average value as the test result. The entire flow test process, from the mixing of the mortar with water to the end of the diameter measurement, should be controlled within 6 min.

#### 2.3.2. Mechanical Strength

The test of mechanical strength in this experiment was in accordance with GB/T 17671-1999 [28]. The compressive strength and flexural strength of specimens at 7, 28 and 90 days were measured by a Universal Testing Machine (Wance, Shenzhen, China) at a loading speed of 2.4 kN/s and 0.05 kN/s, respectively. Specimen sizes for tests of flexural strength and compressive strength were 40 mm × 40 mm × 160 mm and 40 mm × 40 mm × 40 mm, respectively. There were three flexural strength test specimens in each group, and the average of the three specimen test results was calculated, which was the result of the flexural strength test. If the test result was more than 10% of the average value, the value was discarded and the average value of the remaining values was taken as the result. The flexural strength test was conducted using the fracture of the six test pieces as compressive strength test specimens, and we calculated the average value of the test results of the six test pieces, and the average value was the compressive strength test results. If there was more than 10% of the average value in the test results, the value was discarded, and the average of the remaining values was taken as the result.

#### 2.3.3. Impact Resistance

**Figure 6:**
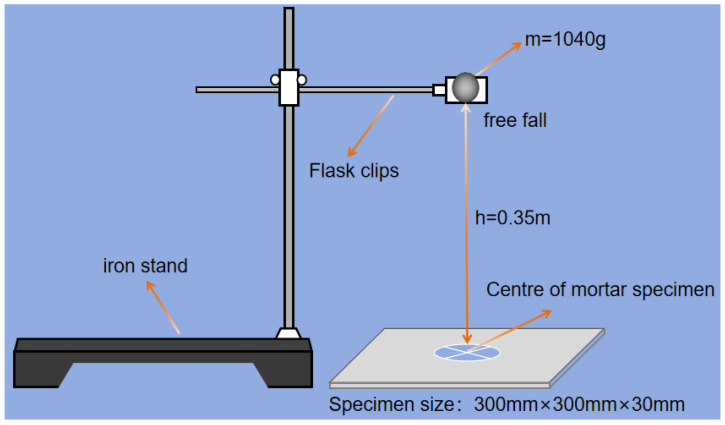
Schematic diagram of the impact test.

The test method of impact resistance referred to GB/T 38494-2020 [29]. The specimen for the impact resistance test was a plate of 300 mm × 300 mm × 30 mm. When the specimen was cured for 28 days, the impact test was carried out on the specimen using the falling ball method. The schematic diagram of the impact test is shown in Figure 6. The height of the falling ball was fixed to fix the impact energy, and the impact strength of the specimen was used as an index for evaluating the impact resistance of the specimen. The first visible cracks that appeared in the impact process of the specimen were taken as the number of initial impacts N_0_, and the number of impacts when the specimen was damaged or a crack width greater than 3 mm was taken as the number of final impacts N_X_. Impact energy dissipation and impact strength were calculated according to Equations (1)–(3):

(1) $$W_{0}=N_{0}\mathrm{mgh}$$

(2) $$W_{x}=N_{x}\mathrm{mgh}$$

(3) $$S=\frac{W_{x}}{t^{2}}$$

where W_0_ and W_x_ refer to the impact energy consumption of initial and final crack(J), respectively; N_0_ and N_x_ refer to the impact number of initial and final crack, respectively. m was the mass of the steel ball (g). In this test, m was 1040 g; h refers to the drop height (m). In this test, h was 0.35 m; g refers to the acceleration of gravity (m/s^2^). In this test, g was 9.81 m/s^2^; S refers to the impact strength (J/mm^2^); and t refers to thickness of the specimen (mm).

#### 2.3.4. Abrasion Resistance

**Figure 7:**
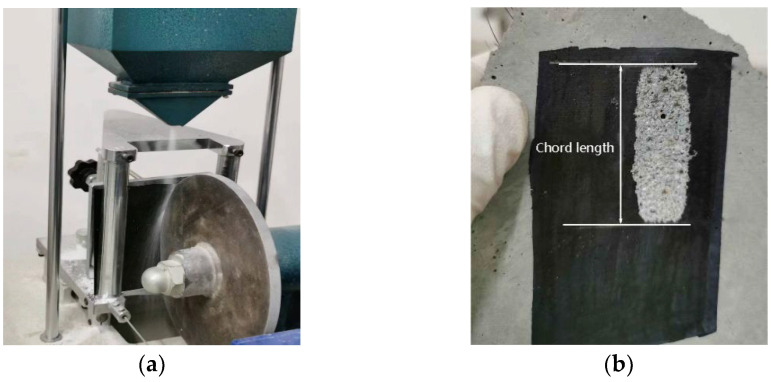
Abrasion resistance tester and tested sample: (**a**) Abrasion resistance tester; (**b**) tested sample.

The abrasion resistance test of the hardened mortar after 28 days was carried out with reference to GB/T 3810.6-2016 [30]. An abrasion resistance tester and test samples are shown in Figure 7, and the feeding rate of the abrasive was 100 ± 10 g/100 r. Vernier calipers were used to measure the chord length of the abrasion pit after 150 r of the friction steel wheel. The mass of the test block was measured before and after the abrasion test, and the mass loss of the test block before and after the experiment was used to express the abrasion mass. The abrasion resistance of the test block was characterized by the volume of the abrasion pit, which was calculated according to Equations (4) and (5):

(4) $$V=\left(\frac{\pi \cdot \alpha }{180}-\sin\alpha \right)\times \left(\frac{h\times d^{2}}{8}\right)$$

(5) $$\sin\frac{\alpha }{2}=\frac{L}{d}$$

where V refers to abrasion volume (mm^3^); L refers to the chord length (mm); α refers to the angle formed by the chord length and the center of the rotating steel wheel (°); h refers to the thickness (mm), which in this test h was 10 mm; and d refers to the diameter of the rotating steel wheel (mm), which in this test h was 200 mm.

#### 2.3.5. Microstructure Testing Methods

The microscopic morphology of mortar was obtained by scanning electron microscopy (SEM, JSM-5900, Japan Electronics Corporation, Osaka, Japan). The effect of waste rock wool on the hydration of cement was investigated by thermogravimetry-differential scanning calorimetry (TG-DSC, STA 449 F1, Netzsch, Selb, Germany). The effect of waste rock wool on cement hydration products was studied using an X-ray diffractometer (XRD, 0031D max/RB, Rigaku Corporation, Tokyo, Japan). The influence of waste rock wool on the total heat release and heat release rate of cement was studied using a cement isothermal calorimeter (Calmetrix, Boston, MA, USA).

## 3. Results and Discussion

### 3.1. Slurry Fluidity

As shown in Table 5, waste rock wool reduced the fluidity of the mortar, which gradually decreased with the increase in the dosage. When the dosage was 20%, the fluidity decreased from 210 mm to 189 mm, a decrease of 10.00%, but the fluidity of the mortar still met the requirements of the actual construction. Waste rock wool leads to the reduction of mortar fluidity mainly due to two factors: on the one hand, the waste rock wool has a high water-absorption rate, and with the increase in the dosage, its water-absorption ability was more and more obvious [31]. On the other hand, the fiber morphology of the waste rock wool makes it interlaced, forming a three-dimensional network system, which increases the resistance to the flow of the slurry.

### 3.2. Mechanical Strength

**Figure 8:**
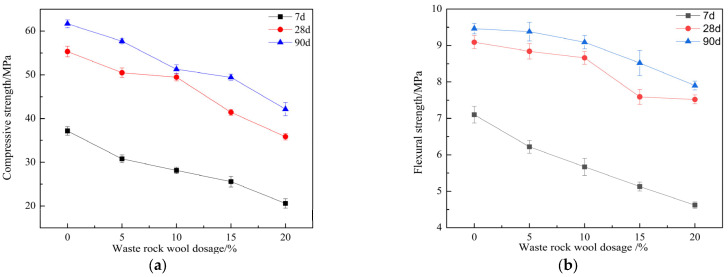
Effect of waste rock-wool dosage on mechanical strength: (**a**) Compressive strength; (**b**) Flexural strength.

Figure 8 shows the compressive strength and flexural strength of mortar with waste rock wool at different ages. Figure 8a reflects the decreasing compressive strength of mortar at 7, 28 and 90 days with the increase in waste rock-wool dosage. The compressive strength of the mortar decreased the most when the dosage of waste rock wool was 20%, and the decrease in compressive strength decreased with the extension of the age period. The 7 days of compressive strength of the mortar decreased from 37.17 MPa to 20.6 MPa (a decrease of 44.58%), the 28 days of compressive strength decreased from 55.32 MPa to 35.85 MPa (a decrease of 35.20%), and the 90 days of compressive strength decreased from 61.67 MPa to 42.15 MPa (a decrease of 31.66%).

In general, the increase in the packing density of cementitious materials in mortar has a positive effect on its mechanical strength. The compressive strength showed a decreasing trend, and as the dosage of waste rock wool increased, the proportion of cement decreased, the dosage of cement hydration products decreased, and the internal structure of the mortar was loosened. The negative effect on the compressive strength was more significant—the combined performance of the compressive strength of the mortar was reduced. However, the increase in compressive strength of the mortar increases with age, which indicates that the detrimental effect of spent rock wool on the later strength of the mortar decreases. This was due to the high water absorption of waste rock wool, which reduces the early hydration rate, and in the later stages of the hydration reaction, waste rock wool can release the absorbed water, which has the effect of internal curing. Klyuev [18] reported similar research results.

Figure 8b demonstrates similar results to the compressive strength, where the flexural strength of the mortar shows a decreasing trend with the increase in the dosage of waste rock wool. The greatest decrease in flexural strength was observed when the waste rock-wool dosage was 20%, and the 7, 28 and 90 days of flexural strength were reduced by 34.93%, 17.27% and 17.19%, respectively. At the age of 28 days, the reduction of the flexural strength of the mortar decreased, the reduction of the flexural strength of the mortar was smaller when the waste rock-wool dosage was less than 10%, and the reduction of the flexural strength continued to increase when the waste rock-wool dosage was more than 10%. When the dosage of waste rock wool was 10%, the flexural strength decreased from 9.09 MPa to 8.66 MPa, which was only 4.73% lower. In order to achieve the requirement of 28 days of flexural strength not less than 8 MPa, the dosage of waste rock wool should be controlled within 10%. At the age of 90 days, the change rule of mortar flexural strength with the dosage of waste rock wool was consistent with the change rule of 28 days of flexural strength with the dosage of waste rock wool. At the age of 90 days, the change rule of mortar flexural strength with the dosage of waste rock wool was consistent with the change rule of 28 days of flexural strength with the dosage of waste rock wool. When the dosage of waste rock wool was 10%, the flexural strength of mortar decreased from 9.54 MPa to 9.09 MPa, which was a decrease of 4.75%.

### 3.3. Toughness

#### 3.3.1. Flexural to Compressive Ratio

In this paper, the flexural and compressive ratio was used to characterize the toughness of cementitious materials, and the flexural and compressive ratio data of 7-, 28- and 90-day ages of waste rock-wool mortar are shown in Table 6. Waste rock wool improves the 7, 28 and 90 days of flexural compression ratios of mortar, which indicates that the waste rock wool has a certain toughening effect. From the data in Table 6, it can be seen that the flexural and compressive ratio of the mortar increases with the dosage of waste rock wool, with an optimal dosage of 20%. At the age of 7 days, the flexural and compressive ratio of mortar increased from 0.191 to 0.224—an increase of 17.28%. At the age of 28 days, the flexural and compressive ratio of mortar increased from 0.164 to 0.210—an increase of 28.05%. At the age of 90 days, the flexural and compressive ratio of mortar increased from 0.155 to 0.187—an increase of 21.13%.

#### 3.3.2. Impact Resistance

The increase in the flexural and compressive ratio of the mortar was due to the fact that the reduction in the compressive strength of the mortar was greater than the reduction in the flexural strength. It was not reasonable to express the toughening effect of waste rock wool by the flexural and compressive ratio alone. Therefore, this research further explored the effect of waste rock wool on the toughness of mortar using the falling ball method.

As can be seen from the data in Table 7, waste rock wool reduced the number of initial and final cracking impacts on the mortar specimens. Increasing the dosage of waste rock wool reduced the impact of initial cracking effects from 16 to 2 (a reduction of 87.50%) and the number of final cracking effects from 60 to 25 (a reduction of 58.33%). The impact strength of the specimen with the increase in waste rock-wool dosage also has a similar rule of change: in 20% of the dosage, the impact strength decreased from 53.56 J/cm^2^ to 22.32 J/cm^2^ (a decrease of 58.33%), which indicates that the waste rock wool weakened the impact resistance of the mortar. Thus, the larger the dosage, the more significant the weakening effect. Waste rock wool plays a role in bridging cracks and preventing microcracks from sprouting and expanding inside the matrix, but its adverse effect on the internal structure of the material was more significant and therefore shows a negative effect on the impact resistance.

**Figure 9:**
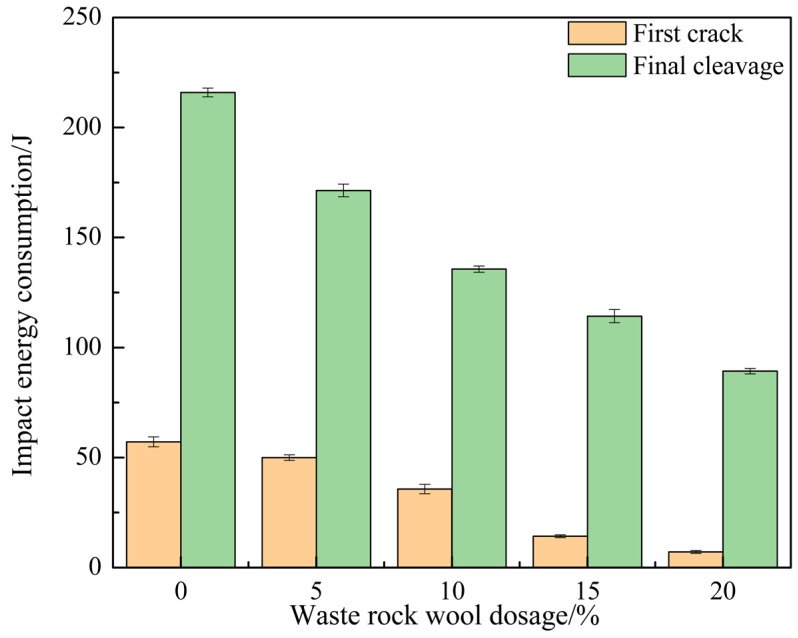
Effect of waste rock wool on total mortar impact energy consumption.

Figure 9 shows the effect of waste rock wool on the total impact energy consumption of mortar. With the increase in waste rock-wool dosage, the total impact energy consumption of mortar at the first and final cracking decreases at the same time. The differences between the two also decreased; the differences were 157.12 J, 121.41 J, 99.98 J, 99.99 J, and 82.13 J. The total energy difference between the initial and final cracking impacts was reduced to the lowest value at a 20% waste rock-wool dosage, indicating that the addition of waste rock wool reduced the toughness of the mortar.

### 3.4. Abrasion Resistance

The incorporation of waste rock wool improved the abrasion resistance of the mortar to a certain extent, and the specific test data are shown in Table 8. The optimum dosage of waste rock wool was 5%, at which time the abrasion volume and abrasive mass of the mortar were 376 mm^3^ and 0.85 g. This was reduced by 24.80% and 29.75%, respectively, compared with R0. The abrasion resistance of the specimens decreased continuously after the dosage of waste rock wool was higher than 5% (except for R15). Because R15 has a uniform distribution of fibers in the abrasion region without agglomeration, its abrasion volume and abrasion mass were instead lower compared to R10. In contrast, the phenomenon that the mortar’s chord length of abrasion volume and abrasion volume were lower than that of the control group while the abrasion mass was higher than that of the control group at 10% dosing was attributed to the increase in abrasion mass due to the shedding of steel fiber in the blended fibers during the abrasion test.

### 3.5. Heat of Hydration

**Figure 10:**
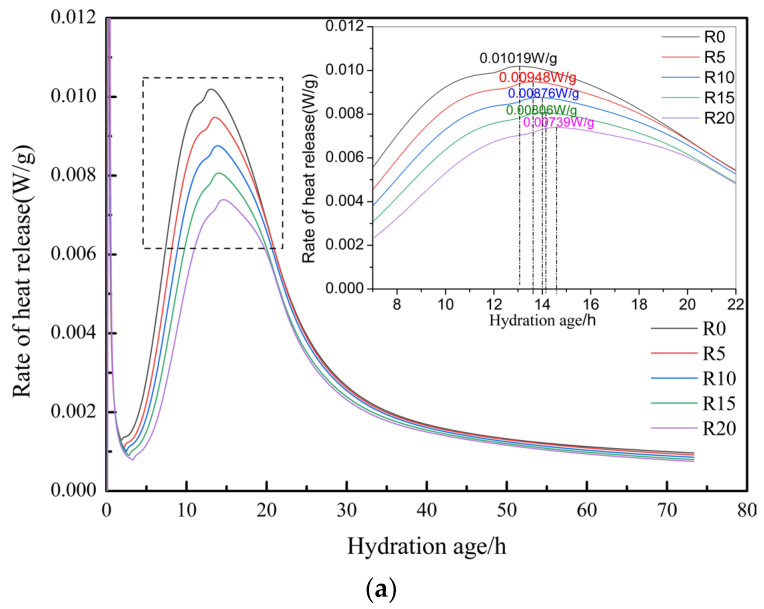

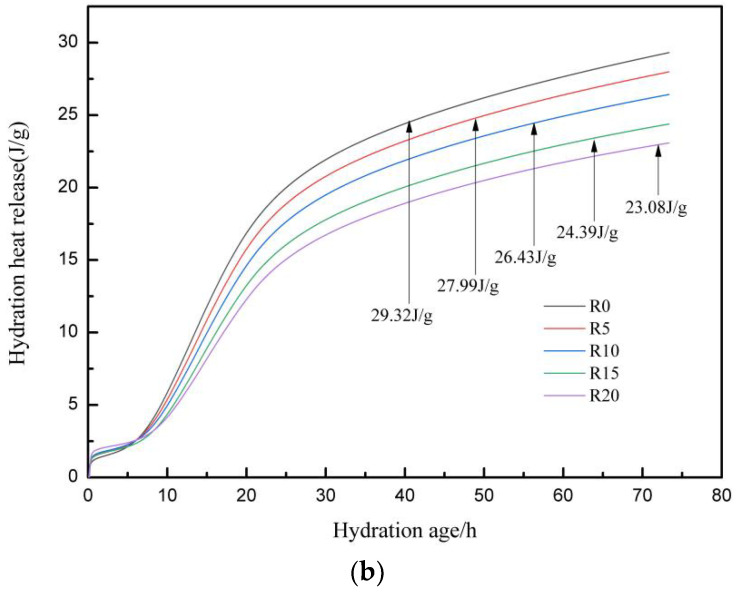
Effect of waste rock-wool dosing on heat of hydration: (**a**) Exothermic rate of hydration; (**b**) Total exothermic hydration.

The hydration process of cement not only involved the generation of hydration products but was also accompanied by the release of a large dosage of heat. The dosage of heat released and the hydration rate of cement hydration were closely related to its hydration degree, so the hydration rate and the heat of hydration can be used to characterize the hydration degree of cement. As can be seen in Figure 10a, the waste rock wool delayed the emergence of the hydration-induced period of the composite slurry. Compared with R0, the dosage of 5%, 10%, 15% and 20% of waste rock wool delayed the appearance of the hydration exothermic peak by 0.55 h, 0.83 h, 1.04 h and 1.59 h, respectively. In the second hydration peak, the shoulder associated with the aluminate hydration reaction and the eventual formation of calculite was also observed [32]. This indicates that the role of waste rock wool in the system was dominated by physical filling, and the increase in the dosage of waste rock wool leads to a decrease in the percentage of cement in the cementitious material, and therefore an increase in the effective water/cement ratio in the system, leading to a decrease in the concentration of calcium ions in the pore solution and therefore an increase in the time required to reach the supersaturated state [33]. The peak of the hydration rate decreases continuously with the increase in waste rock-wool dosage, which was attributed to the dilution effect of waste rock wool that reduces the dosage of C_3_A and C_3_S in the cementitious system, thus slowing down the hydration process [34].

The hydration exotherm of the cementitious material system with different dosages of waste rock wool is shown in Figure 10b, and the dosage of waste rock wool reduces the hydration exotherm of the system. Compared to R0, the exothermic heat of hydration was reduced by 4.54%, 9.86%, 16.81% and 21.28% at dosages of 5%, 10%, 15% and 20%, respectively. At 5% and 10% dosages, the decrease in exothermic capacity is lower than the dosage of waste rock wool, while the two dosages of 15% and 20% lead to a greater decrease in exothermic capacity than the dosage of waste rock wool, which indicates that waste rock wool has a promoting effect on hydration when the mass fraction is not greater than 10%. This was because the waste rock wool has very low activity and hardly reacts, which can increase the effective water/cement ratio of the system when it replaces cement in mortar with a low dosage, thus promoting the early hydration of cement. When the dosage is greater than 10%, waste rock wool will inhibit the hydration process of the cementitious system, which can be attributed to the high water absorption of waste rock wool. When the dosage is larger, it absorbs more water, resulting in a reduction in the effective water/cementitious ratio of the system, which results in a reduction in the degree of the early hydration of cement.

### 3.6. X-ray Diffraction

**Figure 11:**
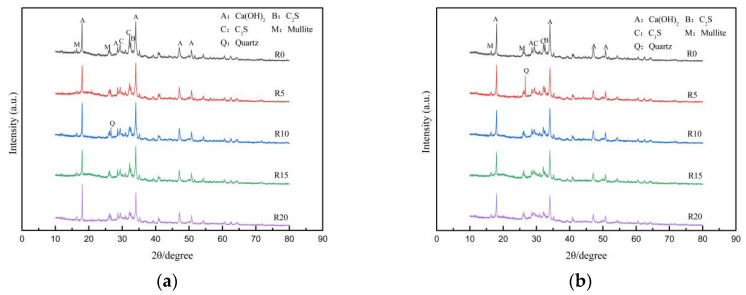
XRD pattern of cement net paste mixed with waste rock wool: (**a**) 3 d; (**b**) 28 d.

Figure 11 shows the X-ray diffraction patterns of the net cement paste at the age of 3 days and 28 days with different waste rock-wool dosages. From the figure, it can be seen that the main crystalline phases in the composite system were AFt, C_2_S, C_3_S, CaCO_3_, Ca(OH)_2_ and quartz, of which C_2_S and C_3_S were the main clinker phases of the cement, AFt and Ca(OH)_2_ were the hydration products, and CaCO_3_ was the result of the carbonation of Ca(OH)_2_. Comparing the control and test groups in Figure 11a,b, it was found that there was no change in the position of the diffraction peaks of the hydration products, which indicated that no new hydration products were generated by the waste rock-wool admixture, and it only affected the relative intensity of the diffraction peaks. From Figure 11a, it can be seen that a 5% to 10% waste rock-wool dosage increased the relative intensity of the diffraction peaks of Ca(OH)_2_ corresponding to 2θ = 34.0°, whereas the relative intensity of the Ca(OH)_2_ diffraction peak gradually decreased at dosages of 15% to 20%. The results are consistent with those of the early hydration analysis of cement.

Comparing Figure 11a,b, it can be found that the relative diffraction peaks of the clinker phases C_3_S and C_2_S decreased significantly with the increase in the age of curing, which indicates that the degree of cement hydration further increased. In contrast, the relative diffraction peak intensity of Ca(OH)_2_ decreased, which indicates that the aluminous and siliceous components in fly ash and silica fume had a secondary hydration reaction with Ca(OH)_2_ and consumed part of Ca(OH)_2_.

### 3.7. TG-DSC Analysis

It was demonstrated that the temperature ranges of 60~150 °C, 390~460 °C and 540~800 °C corresponded to the volatilization of free water and the dehydration of C-H-S gels, the hydrolysis of calcium hydroxide and the decomposition of calcium carbonate, respectively [35,36,37].

**Figure 12:**
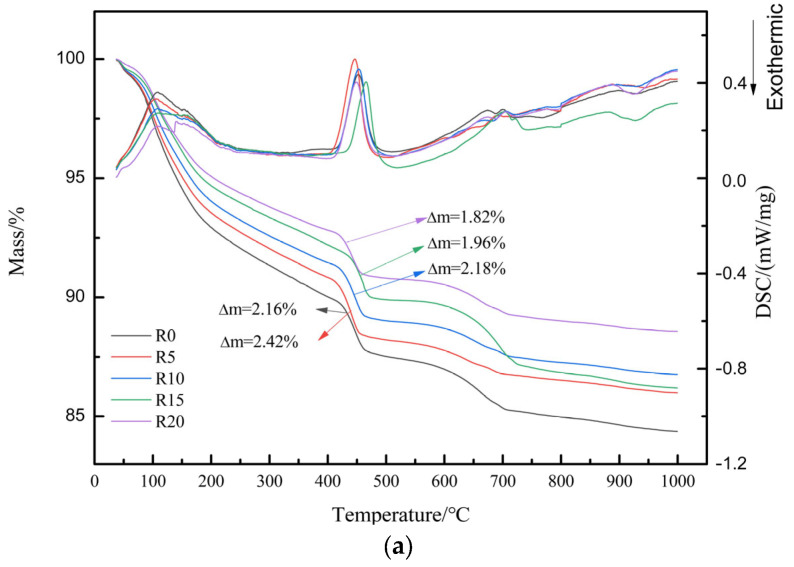

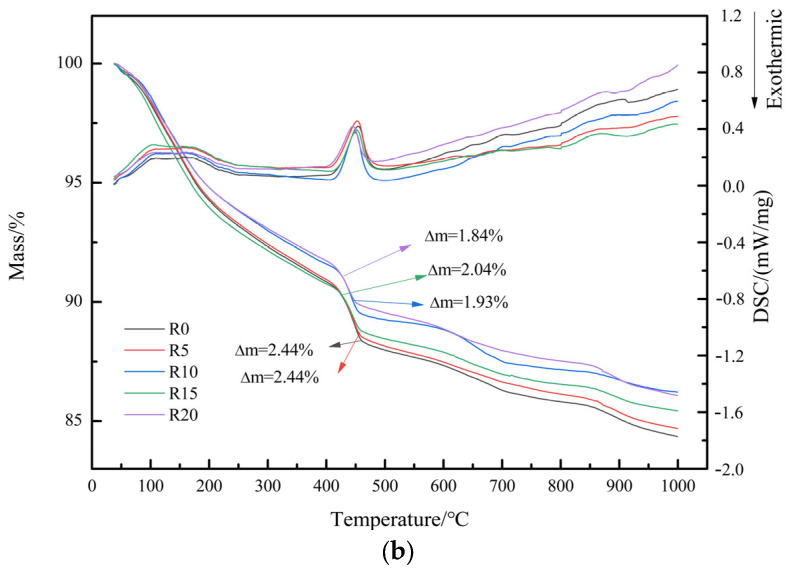
TG-DSC of cement paste mixed with waste rock wool: (**a**) 3 d; (**b**) 28 d.

As can be seen from Figure 12a, the mass loss of calcium hydroxide was 2.16%, 2.42%, 2.18%, 1.96% and 1.82% for R0, R5, R10, R15 and R20 at the age of 3 days, respectively. The content of calcium hydroxide showed an increase and then decrease with the increase in waste rock-wool dosage, which indicated that the waste rock wool promoted the hydration of cement at a low dosage, while the high dosage reduced the degree of cement hydration. This conclusion is consistent with the results of the analysis of the heat of hydration. From Figure 12b, it can be seen that the mass loss of calcium hydroxide was 2.44%, 2.44%, 1.93%, 2.04%, and 1.84% for R0, R5, R10, R15, and R20 at the age of 28 days, respectively. Comparing the mass loss of calcium hydroxide at the age of 3 days and 28 days, it can be seen that the content of calcium hydroxide increases less or even decreases with the increase in age, which indicates that in the hydration of the cementitious material system, fly ash, silica fume and calcium hydroxide undergo a secondary hydration reaction, which further improves the degree of hydration of the cementitious material system, in agreement with the analytical results of the XRD diffractograms.

### 3.8. SEM Analysis

**Figure 13:**
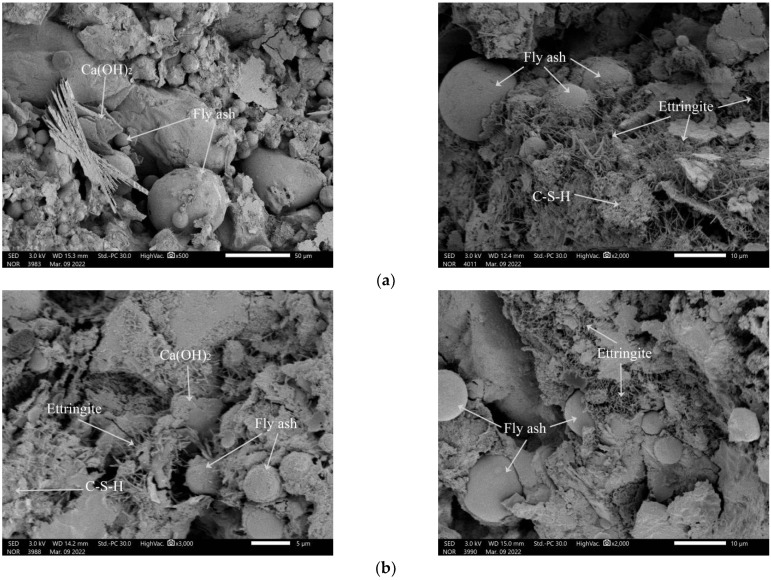
Microscopic morphology of waste rock-wool mortar: (**a**) R0; (**b**) R5.

Figure 13a shows the micro-morphology of hydration products of R0 (without waste rock wool) mortar, and Figure 13b shows the micro-morphology of hydration products of R5 (contains 5% waste rock wool) mortar. As can be seen in the figure, the hydration product type of the mortar remains the same, regardless of the presence or absence of waste rock wool, and C-S-H gel, layered Ca(OH)_2_, ettringite and spherical fly ash particles could be clearly observed in the SEM image of both groups of mortars.

**Figure 14:**
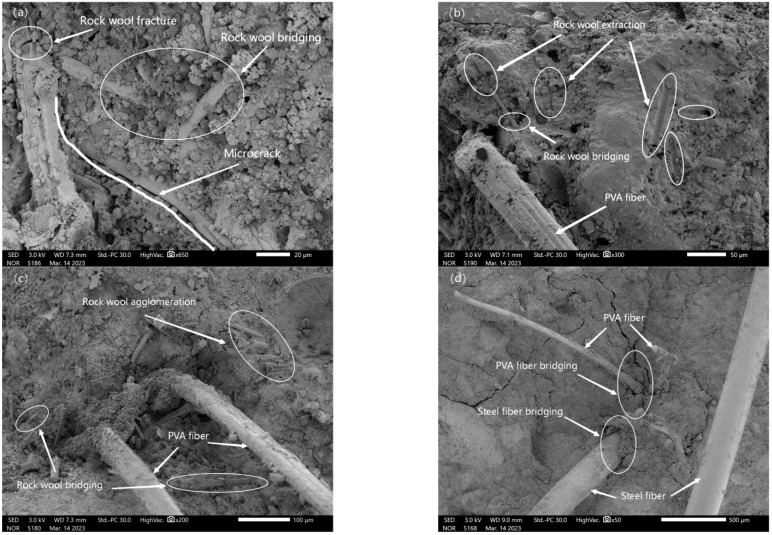
SEM image of multi-scale fiber-reinforced mortar. (**a**) Fibre fracture; (**b**) Fibre extraction; (**c**) Fibre agglomeration; (**d**) Fibre bridging.

The presence of defects such as microcracks inside the cement mortar as well as the self-contraction of the mortar can lead to the generation of microcracks, which are susceptible to stress concentration under loading, leading to the emergence of more and larger cracks [38,39,40]. In cementitious materials, fiber consumes energy through fiber bridging, fiber pullout, fiber breakage and crack deflection to improve the toughness of cement mortar. Different fibers have different effects on the material improvement, and a size of less than 100 µm waste rock wool fills the internal structure and inhibits the initiation and extension of the micro-cracking role. As shown in Figure 14a–c, the waste rock wool with a size less than 100 µm can be used as a filler to fill the internal pores of the mortar. It can also reduce the stress concentration at the crack tip through bridging actions, crack deformations and other action mechanisms. Energy is absorbed to inhibit microcrack expansion and improve the toughness of the mortar through the two destructive forms of tension and pullout (Figure 14a,b). However, when the dosage was too much, the phenomenon of fiber agglomeration occurs, as shown in Figure 14c, and the fiber agglomerates form defects, resulting in the weakening of the toughening effect. Figure 14d shows the effect of PVA fibers and steel fibers. The PVA fibers transmit stress and absorb energy in the form of twisting and deformation or even fracture, while the steel fibers consume energy by overcoming friction with the substrate and pulling it out of the substrate. In addition, in Figure 14b,c, the waste rock wool is randomly distributed around the PVA fibers, while in Figure 14d, it is randomly distributed around steel fibers. This indicates that the waste rock wool, PVA fibers and steel fibers play the roles of crack-blocking and toughening at different structural levels to achieve the layered reinforcement effect.

## 4. Conclusions

In this paper, the effect of waste rock-wool dosage on the workability, strength, toughness, abrasion resistance, and hydration properties of PVA- and steel-fiber hybrid fiber mortar was investigated, and the mechanism of the effect of waste rock-wool dosage on the hybrid fiber mortar was summarized from a microscopic point of view. The research results were as follows:

1. Waste rock wool reduces the flexural strength and compressive strength of the mortar, and in the range of the selected dosage, the larger the dosage of waste rock wool, the greater the reduction in strength. However, with the increase in age, the effect of the reduction of strength was gradually reduced. When the dosage of waste rock wool was 10%, the 28 days of flexural and compressive strengths were reduced by 4.73% and 10.59%.
2. The effect of waste rock wool on the flexural-to-compressive ratio and impact toughness varied. With the increase in waste rock-wool dosage, the flexural-to-compressive ratio of the mortar at all ages increased, and the maximum value of the flexural-to-compressive ratio of the mortar at 28 days under 20% dosage was 0.210, which increased by 28.05%. The impact toughness decreased with the increase in the dosage, and the impact strength of the mortar at 20% doping was 22.32 J/cm^2^, which decreased by 58.33%. Multi-scale hybrid combinations of waste rock wool, polyvinyl alcohol fibers and steel fibers were able to achieve toughening effects at different structural levels.
3. When waste rock wool was used as cementitious material, it consumed waste rock wool and reduced the dosage of cement and auxiliary cementitious materials. This is in line with the concept of green development. Waste rock wool has low activity or even no activity and can only play the role of physical filler. Therefore, in order to maintain high mechanical strength, the amount of waste rock wool should not be higher than 10%.

## Figures and Tables

**Table 1:** Physico-mechanical properties of Portland cement.

Grade	Density (kg/m^3^)	Specific Surface Area (m^2^/kg)	Water Requirement of Normal Consistency (wt%)	Initial Setting Time (min)	Final Setting Time (min)	Flexural Strength (MPa)		Compressive Strength (MPa)	
						3 d	28 d	3 d	28 d
P·II 52.5	3060	372	28	135	261	5.10	8.15	30.75	54.04

**Table 2:** Chemical compositions of the three cementitious materials.

	SiO_2_	CaO	Al_2_O_3_	Fe_2_O_3_	SO_3_	MgO	K_2_O	LOI
Fly ash (%)	50.87	3.06	32.42	7.56	0.37	0.78	1.84	1.84
Cement (%)	20.87	63.66	4.42	2.89	2.10	0.94	0.51	3.30
Silica fume (%)	92.88	0.06	0.62	0.28	-	0.02	0.03	3.85

**Table 3:** Composition of waste rock-wool particle size intervals.

Particle size range/μm	<3	3–16	16–32	32–45	45–80	80–100	>100
Waste rock wool	2.02	21.35	25.85	12.27	16.13	7.25	15.13

**Table 4:** Characteristic parameters of fibers.

Fiber	Diameter (μm)	Lengths (mm)	Tensile Strength (MPa)	Elongation at Break (%)	Modulus of Elasticity (GPa)	Densities (g/cm^3^)
PVA	50	6	1600	7.0	35	1.27
Steel	220	13	≥2000	10	220	7.80

**Table 5:** Mortar ratios and fluidity.

Mix No	W/C	Cement (%)	Waste Rock Wool (%)	Fly Ash (%)	Silica Fume (%)	PVA Fiber (%)	Steel Fiber (%)	Fluidity (mm)
R0	0.5	70	0	25	5	0.09	0.13	210
R5	0.5	65	5	25	5			205
R10	0.5	60	10	25	5			199
R15	0.5	55	15	25	5			194
R20	0.5	50	20	25	5			189

Note: Rx denotes the replacement of cement by rock-wool with a mass fraction of x on the basis of the base proportion of cementitious materials; Fiber dosage is (mortar specimen) volume dosage.

**Table 6:** Flexural and compressive ratio of mortars.

Mix No.	7 d	28 d	90 d
R0	0.191	0.164	0.155
R5	0.202	0.173	0.163
R10	0.201	0.175	0.177
R15	0.201	0.183	0.173
R20	0.224	0.210	0.187

**Table 7:** Effect of waste rock wool on the impact resistance of mortar.

Mix No.	Number of First Crack Impacts N_0_	Number of Final Crack Impacts N_1_	Impact Strength S/(J/cm^2^)
R0	16	60	53.56
R5	14	48	42.85
R10	10	38	33.92
R15	4	32	28.57
R20	2	25	22.32

**Table 8:** Effect of waste rock wool on abrasion resistance of mortar.

Mix No.	Chord Length of Abrasion Volume (mm)	Abrasion Volume (mm^3^)	Mass Loss (g)
R0	39.0	500	1.21
R5	35.5	376	0.85
R10	37.5	444	1.29
R15	35.0	361	0.89
R20	40.5	561	1.34

## Data Availability

The original contributions presented in the study are included in the article, further inquiries can be directed to the corresponding authors.
